# Molecular ultrasound imaging of neutrophil membrane-derived biomimetic microbubbles for quantitative evaluation of hepatic ischemia-reperfusion injury

**DOI:** 10.7150/thno.57794

**Published:** 2021-05-08

**Authors:** Zheng Zhang, Xiaoyan Miao, Weifeng Yao, Jie Ren, Chaojin Chen, Xiang Li, Jing Yang, Yujia You, Yuejun Lin, Tinghui Yin, Ziqing Hei

**Affiliations:** 1 Department of Anesthesiology, The Third Affiliated Hospital of Sun Yat-sen University, Guangzhou 510630, China.; 2 Department of Medical Ultrasonic, Laboratory of Novel Optoacoustic (Ultrasonic) imaging, The Third Affiliated Hospital of Sun Yat-sen University, Guangzhou 510630, China.

**Keywords:** biomimetic, microbubbles, molecular ultrasound imaging, neutrophil, hepatic ischemia-reperfusion injury

## Abstract

**Rationale:** Early diagnosis of hepatic ischemia-reperfusion injury (HIRI), the major cause of early allograft dysfunction or primary non-function, is critical in orthotopic liver transplantation. However, liver biopsy is still the primary method for HIRI evaluation in clinical practice despite its numerous complications and shortcomings such as hemorrhage and inaccuracy. Herein, we aimed to develop a non-invasive, highly accurate, and specific method for detecting HIRI.

**Methods:** We developed a top-down and bottom-up strategy to fabricate neutrophil biomimetic microbubbles (MB_neu_). Neutrophil membrane was mixed with liposomes at a defined mass ratio by sonication. The air in the vial was exchanged with perfluoropropane, and then the solution was mechanically vibrated to form MB_neu_.

**Results:** MB_neu_ retained the neutrophil proteins, preferentially targeted inflamed hepatic tissue in a rat model of HIRI, and demonstrated physicochemical properties typical of liposome-based MBs because of its artificial phospholipid content. With MB_neu_ we can quantitively evaluate the severity of HIRI, which is helpful for early diagnosis and the prediction of outcome. In addition, MB_neu_ was shown to be safe and showed no immunogenicity.

**Conclusion:** We demonstrated molecular ultrasound imaging of HIRI with MB_neu_. This new synthesis strategy may be applied to different clinical scenarios using other cell types in the future.

## Introduction

Hepatic ischemia-reperfusion injury (HIRI) is the major cause of morbidity and mortality following orthotopic liver transplantation (OLT) 1. Serious HIRI results in acute and chronic rejection, donor organ shortage, early allograft dysfunction, and primary nonfunction. All such complications are associated with poor clinical outcome 2, 3. Hence, early diagnosis and intervention of HIRI might improve prognosis 4. Liver biopsy is currently considered as the gold standard for HIRI diagnosis. However, it cannot be used for routine and dynamic monitoring of HIRI owing to its invasiveness and sampling error 5. Biochemical parameters such as alanine transaminase (ALT) and aspartate transaminase (AST) are commonly used in clinical practice. However, the morphology and location of HIRI damage cannot be determined based on these parameters alone 6. Imaging methods including magnetic resonance imaging have been used to assess HIRI, but their application remains in the preclinical stage 5-7.

Contrast-enhanced ultrasound (CEUS) combining ultrasound contrast agents (UCAs) and contrast-specific imaging techniques has already become a valuable clinical technique for assessing hepatic perfusion after OLT due to its advantages including no ionizing radiation, real-time detection, and reproducibility 8, 9. As an important branch of CEUS, molecular ultrasound (US) imaging has emerged as a new method for visualizing molecular targets using ligand-functionalized microbubbles (MBs), thus providing anatomical and functional information simultaneously with high sensitivity and specificity 10, 11. Currently, molecular US imaging is widely used in preclinical research and has shown good accuracy 12, 13. Liposome-based MBs functionalized with targeting ligands are the most common type of targeted UCAs in preclinical studies. Our previous research on this imaging technique achieved some progress in HIRI visualization 14. However, single target UCAs with high diagnostic specificity have been demonstrated to provide low sensitivity. Moreover, the process of targeting ligand functionalization might lead to immunogenicity, and the resulting risk of allergies would limit clinical translation of these UCAs 15. Recently, several biomimetic cell membrane-derived nanoparticles have garnered interest as theranostic systems for diverse diseases with advantages such as biocompatibility, long retention time, and naive targeting ability 16-18. This approach is worth considering for developing non-immunogenic targeted UCAs for HIRI visualization.

The pivotal role of neutrophils in HIRI has been extensively recognized. Many studies have demonstrated that liver injury and inflammation are alleviated after HIRI either by elimination of neutrophils or suppression of their function 2, 19. An inflammatory reaction is initiated in the ischemia phase that persists throughout the reperfusion period, which increases the expression of mediators such as cytokines, chemotactic factors, and adhesion molecules that recruit large numbers of neutrophils to the inflammatory site. Tracking neutrophil infiltration is a good way to determine the distribution and severity of the inflammatory response. Therefore, we aimed to endow UCAs with chemotactic and adhesion properties similar to those of neutrophils for HIRI visualization.

**Figure 1:**
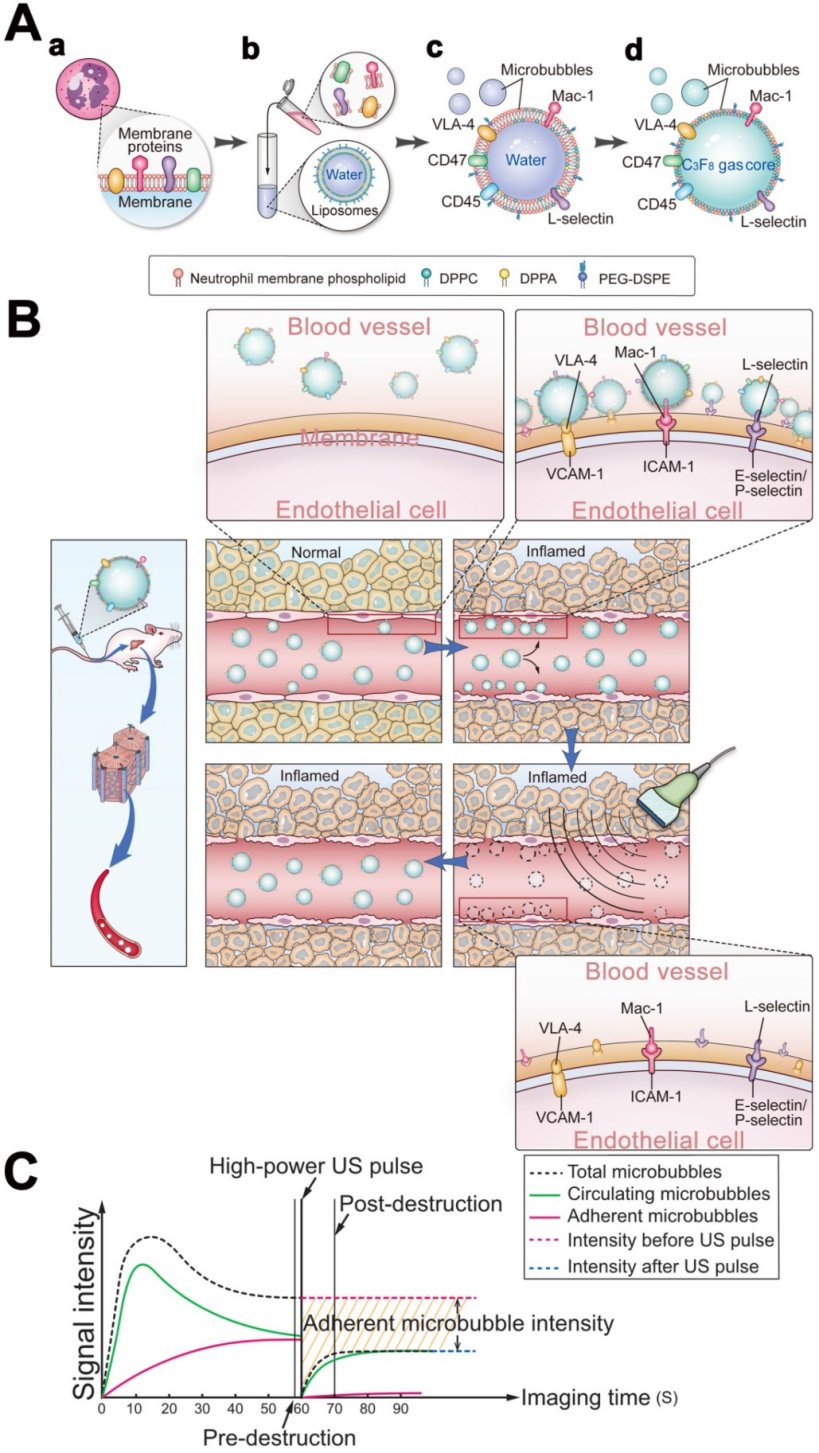
** Schematic illustration of the preparation of MB_neu_ and the principles of the destruction-replenishment method for quantitative targeted US imaging. (A)** Synthesis of MB_neu_. Peripheral neutrophils isolated from rats (a) were mixed with synthetic liposomes (b). The neutrophil membrane was hybridized with the liposomes (c) and then the gas was exchanged to obtain MB_neu_ (d). **(B)** Basis of the destruction-replenishment method. Neutrophil adhesion receptors expressed on liver sinusoidal endothelial cells (e.g., ICAM-1, VCAM-1, and E-selectin/P-selectin) are significantly upregulated in inflamed tissues after HIRI, resulting in attachment of MB_neu_ to the intrahepatic vessels following intravenous administration. Both attached and freely circulating MB_neu_ were destroyed by a high-power US pulse within the beam elevation of the transducer. Freely circulating MB_neu_ reappeared rapidly but failed to attach to the intrahepatic vessels because the receptors were temporarily blocked. **(C)** Calculation of US signal intensity during the destruction-replenishment method. One minute after intravenous administration, US signals from freely circulating MB_neu_ and attached MB_neu_ were obtained as the first US data. The second US data was acquired ~10 s after the high-power US pulse, thus allowing the freely circulating MBneu to replenish the imaging plane. The US signal from MB_neu_ attached to the molecular target was expressed as the difference in US signal intensity before and after the destruction pulse. NID was calculated as (signal pre-destruction - signal post-destruction) / signal pre-destruction × 100%.

This study describes the development of stable and non-immunogenic neutrophil biomimetic microbubbles for accurate visualization of HIRI (Figure 1).

## Materials and Methods

### Preparation of neutrophil membrane

Whole blood was collected from male Sprague-Dawley rats through the abdominal aortic method using EDTA pre-treated tubes. An equal volume of blood sample and neutrophil separation liquid (Solarbio, China) were gently mixed and then centrifuged at 1000 × *g* for 30 min at room temperature. Two layers of milky white spherical cells were observed, and neutrophils were extracted from the lower layer. Next, red blood cell lysate was added to the neutrophils (3:1 v/v), the sample was mixed for 10 min for hemolysis, and the red supernatant was discarded. Then, 10 mL of washing buffer was mixed with the neutrophils, the samples were centrifuged at 250 × *g* for 10 min, and the supernatant was discarded. The above washing step was repeated and purified neutrophils were obtained. Subsequently, the neutrophils were resuspended in 12 mL of hypotonic lysis solution (30 mM pH 7.5 Tris-HCl, 225 mM D-mannitol, 75 mM sucrose, 0.2 mM EGTA, protease inhibitor, phospholipase inhibitor), transferred to a glass homogenizer, then homogenized 20 times on ice. Next, the solution was transferred to centrifuge tubes and centrifuged at 10,000 × *g* for 30 min. The supernatant was discarded, and the precipitate was resuspended in triple distilled water. Finally, the solution was frozen at -80 °C and the neutrophil membrane was obtained by vacuum lyophilization and then stored at -20 °C.

### Preparation of liposomes

Liposomes were fabricated using a thin-film hydration-sonication method from 1,2-dipalmitoyl-*sn*-glycero-3-phosphocholine (DPPC), 1,2-dipalmitoyl-*sn*-glycero-3-phosphate (DPPA), and 1,2-distearoyl-*sn*-glycero-3-phosphoethanolamine-*N*-[biotinyl(polyethylene glycol)_2000_] (DSPE-PEG_2000_) in PBS. Briefly, phospholipids at a molar ratio of DPPC:DSPE-PEG_2000_:DPPA = 18:1:1 (18 mg DPPC, 3.5 mg DSPE-PEG_2000_, 1 mg DPPA) were dissolved in 4 mL of chloroform that was removed by rotary evaporation to form a thin phospholipid film. The film was then hydrated with 4 mL of phosphate-buffered saline (PBS) and maintained at 60 °C in a shaker-incubator for 30 min to form liposomes. Afterwards, 4 mL of liposomes was sonicated until the solution became clear and then 0.5 mL was transferred to individual vials.

### Preparation and characterization of neutrophil membrane-derived MBs (MB_neu_)

Neutrophil membrane and liposomes were mixed at a defined mass ratio by sonication for 15 s. The air in the vial was exchanged with perfluoropropane (C_3_F_8_), and the solution was mechanically vibrated for 45 s using a shaker to form MBs. The suspension was subsequently washed with PBS by centrifugation (3000 rcf, 2 min) to remove excess cell membrane. Erythrocyte MBs (MB_ery_) were fabricated with addition of erythrocyte membrane instead of neutrophil membrane. Control MBs (MB_con_) were fabricated without addition of cell membrane.

The size and zeta potential of the MBs were measured by dynamic light scattering (DLS). A fluorescence microscopy experiment was performed to confirm the presence of neutrophil membrane in the MB_neu_ shell. The synthetic liposomes were fluorescently labeled by addition of DiO in the lipid mixture before liposome formation, and DiI was used to trace the neutrophil membrane. Furthermore, western blotting was conducted to detect membrane proteins in MB_neu_. Detailed procedures are provided in the **Supplementary Materials**.

### *In vitro* stability and CEUS imaging ability of MB_neu_

The *in vitro* stability and CEUS suitability of MB_neu_ with various mass ratios (neutrophil membrane: synthetic phospholipid = 1:2, 1:5, 1:10, 1:50, 1:100, 1:300, 1:600) and MB_con_ were compared using a custom 2% (w/v) agarose mold described previously^2^. 1 mL of MBs in PBS (5 × 10^5^ MB/mL) was added to the sample well. A clinical US scanner (VINNO70, China) with a broadband high-frequency linear transducer (X4-12L) was used in contrast pulse sequencing mode with the following imaging parameters: frequency, 4.5 MHz; transmission power, -80 dB; mechanical index, 0.04. The focal zone was placed at the center of the sample well. A horizontal imaging plane through the agarose mold was used. US images were recorded from each sample after gentle stirring with a glass rod every 10 min.

### Animals

Male Sprague-Dawley rats (10-12 weeks old, 220-250 g) were purchased from the laboratory animal center of Guangdong Province. All protocols involving animals were approved by the local Ethics Committee of the Third Affiliated Hospital, Sun Yat-sen University and were performed in accordance with the guidelines outlined in the National Institutes of Health Guide for the Care and Use of Laboratory Animals.

### Preparation of HIRI animal model

The rats were acclimatized to the housing conditions (room temperature, 24-27 °C) for approximately one week prior to the start of the experiments. A partial (70%, left lobe and middle lobe of the liver) HIRI model was developed as reported previously. Briefly, under aseptic conditions, the abdomen of anesthetized rats was opened along the ventral linea alba, the Glisson system was isolated from the left and middle liver lobes, and an atraumatic microvascular clamp (Fine Science Tools, Canada) was used to obstruct the blood supply to these two lobes. Rats were divided into four groups according to the ischemia induction time: sham (no vasculature occlusion), mild HIRI (30 min), moderate HIRI (60 min), severe HIRI (90 min). At the end of the ischemia period, the clamp was loosened and blood flow was restored. After 3 h of reperfusion, targeted CEUS was performed and the rats were sacrificed for collection of liver and blood. The severity of liver injury was graded using the Suzuki criteria 20. The activities of AST and ALT in serum were measured using a clinical chemistry analyzer (Hitachi 7150). Rat livers were fixed in 4% paraformaldehyde for 24 h. Hematoxylin/eosin (H&E) staining of liver tissue sections (3 mm) was performed after deparaffinization. Three paraffin sections of each sample were used for H&E. Immunohistochemistry was carried out to determine the expression of inflammatory molecular markers. Detailed procedures are provided in the **Supplementary Materials**.

### *In vivo* US imaging

Targeted CEUS was performed using two clinical US imaging systems (RE7, Mindray, China; EPQ7 digital premium US system, PHILIPS, Netherlands) with a high-frequency linear array transducer operating with the following parameters: mechanical index, 0.08; frequency, 10 MHz; imaging depth, 3-4 cm. All imaging settings were kept constant throughout the imaging sessions for all animals. Targeted US images were acquired via a destruction-replenishment method (Figure 1C) as follows: After 60 s of continuous imaging of blood and free MBs, all MBs in the region were destroyed by increasing the mechanical index from 0.08 to 0.24 over 1 s using a “flash” function. Subsequent post-destruction imaging lasted for 10 s, and freely circulating MBs were imaged. (Prototype videos to show perfusion, destruction and reperfusion of microbubbles in HIRI rats' livers from MB_con_ and MB_neu_ in attachments numbered Video 1~4).

An experiment was performed in HIRI rats (n = 6) to explore the *in vivo* stability and imaging ability of MB_con_ and MB_neu_ with various mass ratios of neutrophil membrane to synthetic phospholipid (1:10, 1:50, 1:100). Briefly, 50 μL of each MB formulation (1 × 10^7^ MB/mL) was injected via the tail vein in random order with 30 min between administrations to allow for clearance. After injection of each type of MB, destruction-replenishment imaging was performed as described above.

Another experiment involving the sham group (n = 6) and HIRI rats (n = 8) was performed to compare the targeted imaging ability of MB_con_, MB_neu,_ and MB_ery_. Three MBs with the same volume and concentration were administrated in random order via the tail vein. To investigate the ability of the MB_con_ and MB_neu_ (neutrophil membrane to synthetic phospholipid ratio, 1:50) to distinguish between HIRI of different ischemic times/grades in the animals, sham group (n = 6), mild HIRI (n = 8), moderate HIRI (n = 7), and severe HIRI group (n = 6) were included in the study. The MBs (50 μL of 1 × 10^7^ MB/mL) were administrated in random order via the tail vein. After injection of each type of MB, destruction-replenishment imaging was performed as described above.

### Quantitative analysis of targeted US imaging

All imaging data were analyzed offline in random order as described previously 14. Briefly, the CEUS quantitative analysis software Sonamath (Ambition T.C., China) was used to quantify the targeted US imaging signal from MBs bound to neutrophil adhesion molecules. Images of the regions of interest were generated using pre-destruction contrast frames representing both the attached and free MBs and post-destruction contrast frames representing only the freely circulating MBs. The targeted US signal from the attached MBs was calculated by subtracting the post-destruction signal from the pre-destruction signal. Normalized intensity difference (NID) was used to quantify the imaging signal. NID was calculated as the ratio of the imaging signal intensity of the attached MBs to the imaging signal intensity of total MBs. Images representing the attached MBs are displayed as a color-coded signal overlayed on the B-mode image.

### Biosafety and immunogenicity of MB_neu_

Detailed procedures are provided in the **Supplementary Materials**.

### *In vivo* distribution of MB_neu_

Detailed procedures are provided in the **Supplementary Materials**.

### Circulation time of MB_neu_

Detailed procedures are provided in the **Supplementary Materials**.

### Statistical analysis

All statistical analyses were conducted using SPSS v22.0 (SPSS Inc., USA). Quantitative data are presented as mean ± standard error of the mean. T-test was used to compare two groups. ANOVA was used to compare multiple groups and further pairwise comparison was conducted with the least significant difference method. *P* values (two-tailed) less than 0.05 were deemed to indicate statistically significant differences.

## Results

### Characterization of MB_neu_

**Figure 2:**
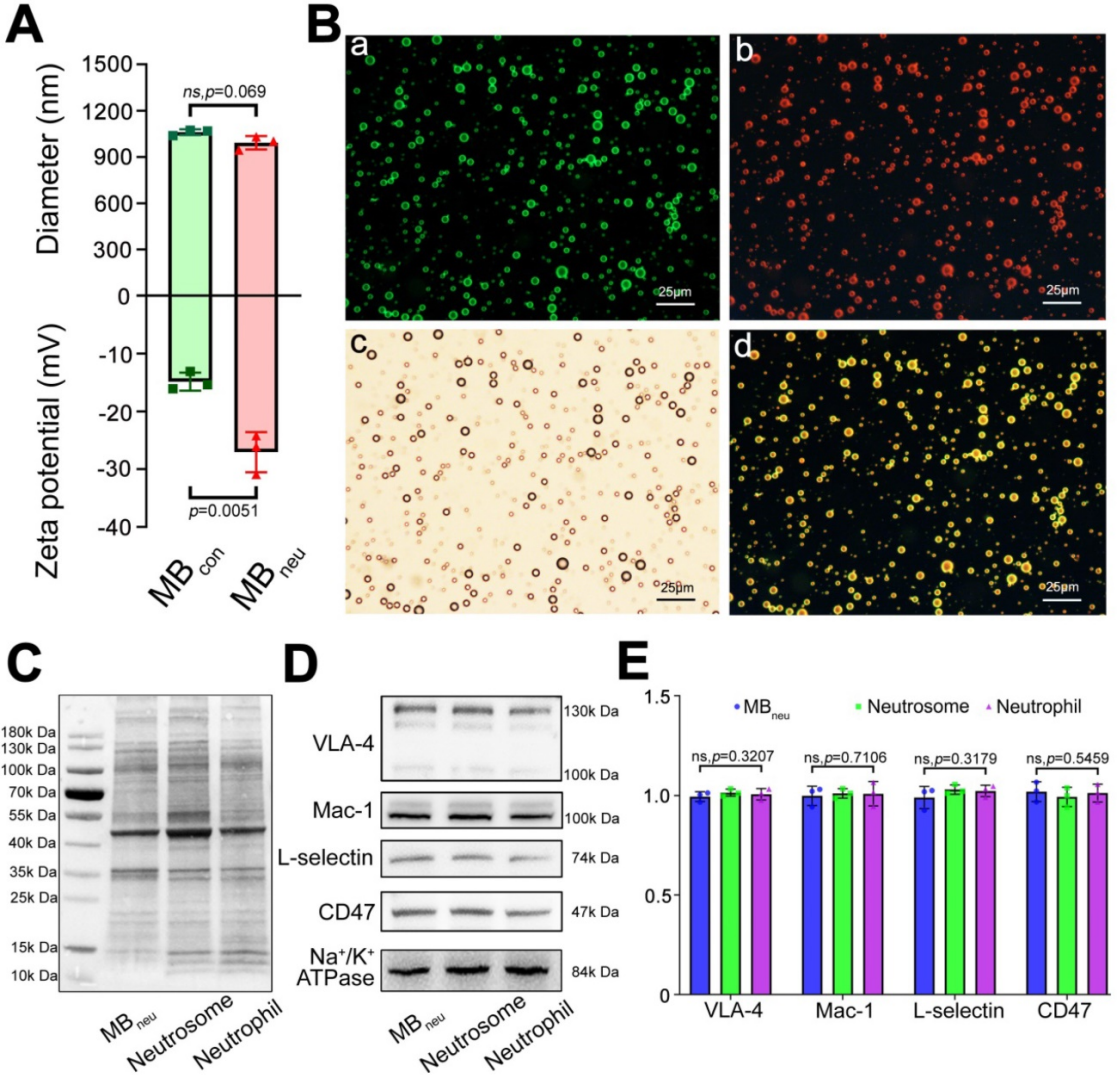
** Characterization of MB_neu_. (A)** Diameters and zeta potentials of MB_con_ and MB_neu_ (n = 3). **(B)** Fluorescence microscopy images of MB_neu_. (a) Liposomes were stained with DiO. (b) Neutrophil membrane was stained with DiI. (c) Merged image. (d) Bright-field image. **(C)** Whole protein and **(D)** targeting ligands for chemotaxis and adherence in MB_neu_, neutrophil membrane (neutrosome), and neutrophils resolved by western blotting. **(E)** Quantitative analysis of D (n = 3).

As shown in Figure 2A, DLS measurements determined that the mean diameter of MB_neu_ was 1.01 ± 0.02 μm, which was similar to that of MB_con_ (1.06 ± 0.02 μm, *P* = 0.069). However, MB_neu_ had a lower zeta potential than MB_con_ (-27.07 ± 3.45 mV vs. -14.87 ± 1.57, *P* = 0.0051), probably due to integration of neutrophil membrane protein into MB_neu_. Representative size and zeta potential distributions are provided in Figure S1.

To validate the successful integration of cell membrane with liposomes in MB_neu_, a fluorescence microscopy study was performed. As shown in Figure 2B, green fluorescence from DiO included in the synthetic liposomes and red fluorescence from DiI used to trace the neutrophil membrane lipids overlapped in the merged image, indicating the successful fabrication of MB_neu_.

Along with phospholipids, neutrophil membrane proteins were integrated into the shell of MB_neu_ (Figure 2C-E). These proteins included targeting ligands for chemotaxis and adherence such as Mac-1, VLA-4, and L-selectin. These ligands are known to specifically bind to inflammatory molecular markers such as ICAM-1, VCAM-1, and P-selectin/E-selectin, which should result in adherence of MB_neu_ to endothelial cells in inflamed hepatic regions.

### Pathological changes and expression of inflammatory molecular markers after HIRI in rats

**Figure 3:**
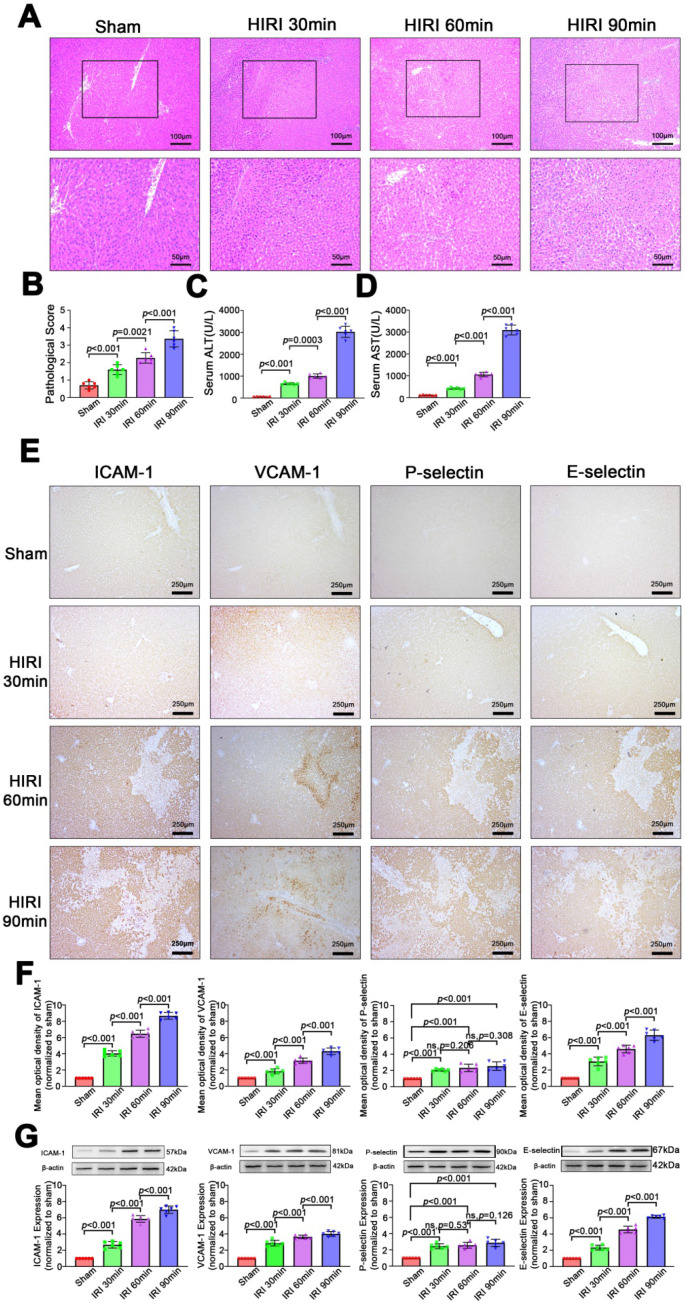
** Morphological and functional changes and expression of inflammatory molecular markers in the liver after HIRI. (A)** H&E-stained sections of liver tissues. **(B)** Pathological score (n = 6). **(C)** Serum ALT activity (n = 6). **(D)** Serum AST activity (n = 6). **(E)** Protein expression of inflammatory molecular markers in liver tissues detected by immunohistochemistry. **(F)** Quantitative analysis of E (n = 6). **(G)** Protein expression of inflammatory molecular markers in liver tissues detected and quantitated by western blotting (n = 6).

As shown in Figure 3A-D, the severity of liver injury, which is assessed based on the pathological score and serum ALT and AST levels, increased with prolongation of the ischemia period (*P* < 0.05) in rats. Similarly, as shown in Figure 3E-G, expression of inflammatory markers (ICAM, VCAM, P-selectin, E-selectin) was low in sham rats. Among HIRI animals, expression of inflammatory markers was higher in the moderate group than in the mild group (*P* < 0.05) and the highest expression of inflammatory markers was in the severe group (*P* < 0.05), demonstrating that expression of inflammatory markers was positively correlated with hepatic pathological lesions.

### Targeted US imaging ability of MB_con_, MB_neu_, and MB_ery_

**Figure 4:**
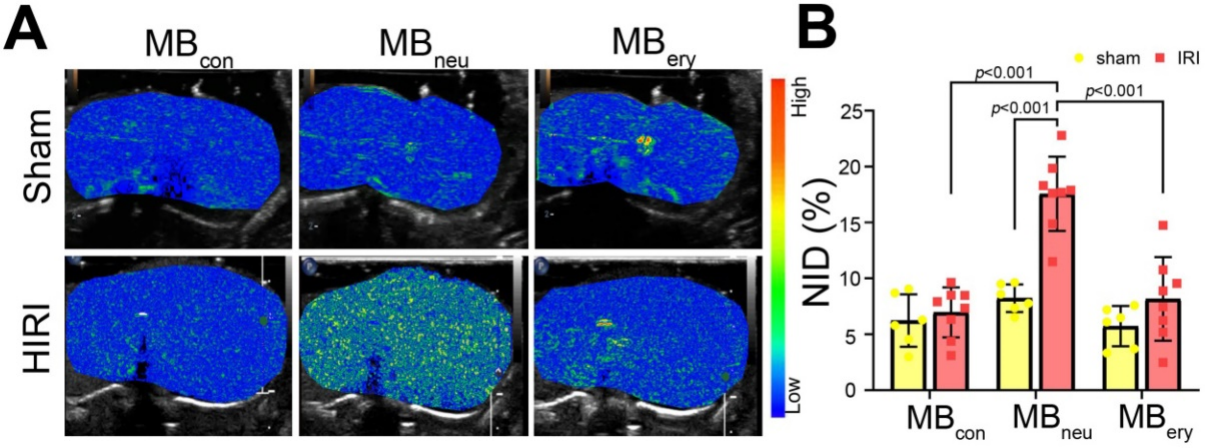
** Targeted US imaging of HIRI by MB_neu_. (A)** Representative targeted US images of MB_con_, MB_neu_, and MB_ery_ in sham and HIRI rats. **(B)** NIDs calculated using the destruction-replenishment method (n = 6 in the sham group, n = 8 in the HIRI group).

To verify the targeted imaging ability of MB_neu_, sham and HIRI rats were intravenously administered MB_con_, MB_neu_, and MB_ery_ in random order (Figure 4A). The HIRI groups showed much greater signal intensity after MB_neu_ administration than the sham group. In addition, a stronger intensity was observed after MB_neu_ administration than after MB_con_ and MB_ery_ administration in HIRI rats. There were also no obvious differences between sham and HIRI groups after MB_con_ and MB_ery_ administration. Moreover, quantitative analysis demonstrated that no significant difference was observed in sham rats after MB_con_ (6.23 ± 2.14%), MB_neu_ (8.23% ± 1.12%), or MB_ery_ (5.74% ± 1.64%) administration (*P* = 0.077). In contrast, a significantly higher NID was observed in HIRI rats after MB_neu_ administration (17.56% ± 3.10%) than after MB_con_ (6.96% ± 2.09%, *P* < 0.001) or MB_ery_ (8.17% ± 3.50%,* P* < 0.001) administration (Figure 4B).

### *In vitro* stability and US imaging ability of MB_neu_

**Figure 5:**
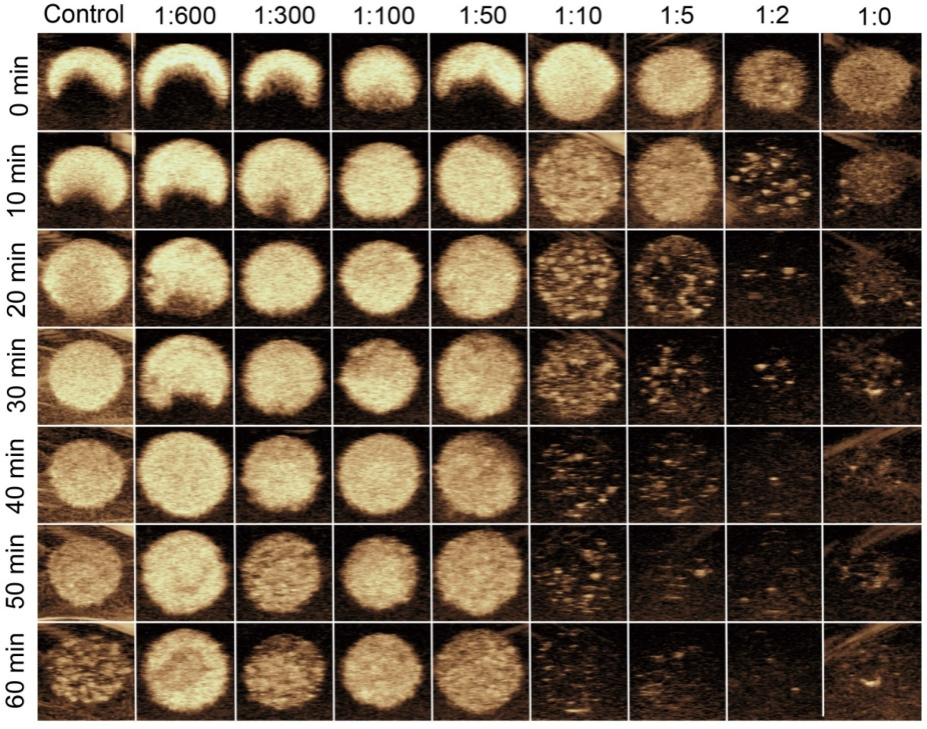
*In vitro* stability and US imaging ability of MB_neu_ with various cell membrane/synthetic phospholipid ratios.

Representative longitudinal CEUS images of MB_neu_ with various mass ratios of neutrophil membrane to synthetic phospholipid are shown in Figure 5. The results demonstrated that when the ratio was less than or equal to 1:50, the US signal intensities were stable up to 60 min. In contrast, the signal intensities dropped substantially when the ratio was greater than 1:50, which indicated that the stability of MB_neu_ decreased with increasing amount of neutrophil membrane.

### *In vivo* stability and US imaging ability of MB_neu_

**Figure 6:**
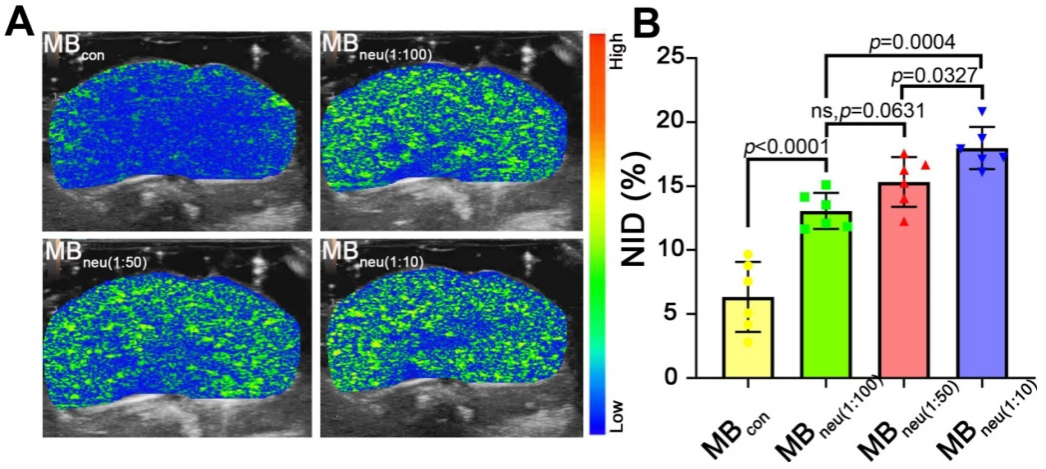
***In vivo* US imaging ability of MB_neu_ with various cell membrane/synthetic phospholipid ratios. (A)** Representative targeted US images of MB_neu_ in HIRI rat livers. **(B)** NIDs calculated using the destruction-replenishment method (n = 6).

To acquire targeted US images, MBs with various mass ratios of neutrophil membrane to synthetic phospholipid (1:10, 1:50, 1:100) were administered to HIRI rats through the tail vein. As shown in Figure 6, a much stronger US signal intensity was observed in rats that received MB_neu_ compared with those that received MB_con_. Quantitative analysis of the US signal intensities as NIDs showed consistent results (*P* < 0.0001, Figure 6B). Furthermore, the targeted US signal intensity after administration of MB_neu_ was greater for a neutrophil membrane to synthetic phospholipid ratio of 1:10 (17.98% ± 1.50%) than 1:50 (15.33% ± 1.77%, *P* = 0.033) and 1:100 (13.07% ± 1.29%, *P* < 0.001). However, the signal intensities for MB_neu_ with ratios of 1:50 and 1:100 were not statistically different (*P* = 0.063). Therefore, we chose the MB_neu_ formulation with a ratio of 1:50 for subsequent experiments. The circulation time of this MB_neu_ was measured to be 6-7 min, which was similar to that of MB_con_ (Figure S2).

### Quantitative US imaging of HIRI severity by MB_neu_

**Figure 7:**
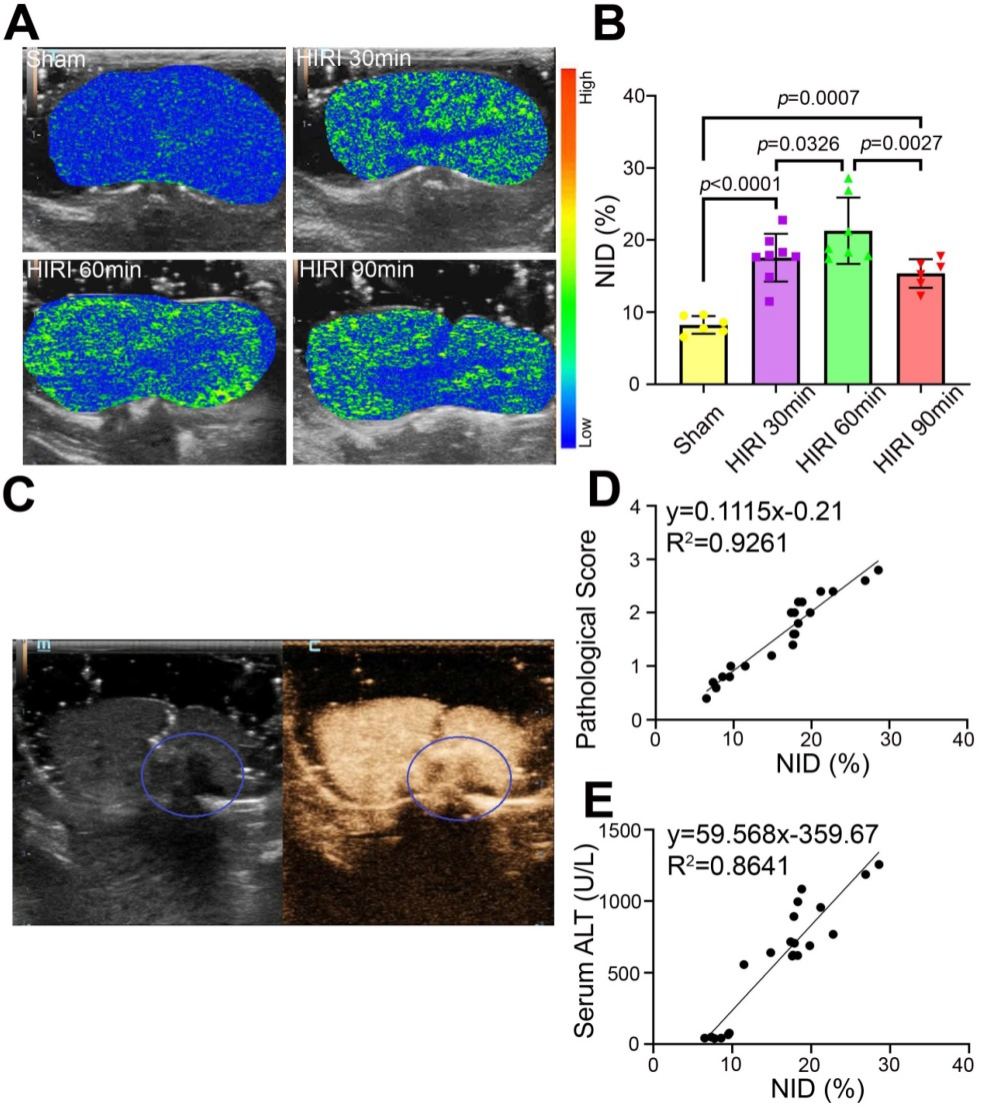
** Targeted US imaging of HIRI severity by MB_neu_. (A)** Representative targeted US images of MB_neu_ in rats with varying severity of HIRI. **(B)** NIDs calculated using the destruction-replenishment method (n = 6 in the sham group, n = 8 in the mild HIRI group, n = 7 in the moderate HIRI group, n = 6 in the severe HIRI group). **(C)** Representative CEUS image of a rat in the severe HIRI group. The blue circle indicates the filling defect in the liver. **(D)** Correlation analysis between pathological score and NID for MB_neu_ in the sham, mild HIRI, and moderate HIRI groups (*R*^2^ = 0.9261, *P* < 0.0001). **(E)** Correlation analysis between serum ALT and NID for MB_neu_ in the sham, mild HIRI, and moderate HIRI groups (*R*^2^ = 0.8641, *P* < 0.0001).

Further targeted US imaging experiments were performed to distinguish mild, moderate, and severe HIRI. Notably, the strongest signal intensity after administration of MB_neu_ was observed in the moderate HIRI group (Figure 7A). The signal intensity was weaker in the mild and severe HIRI groups. Quantitatively, the NID of the moderate HIRI group (21.19% ± 4.26%) was significantly higher than those of the mild (17.56% ± 3.10%, *P* = 0.033) and severe (15.33% ± 1.77%, *P* = 0.003) HIRI groups (Figure 7B). The NIDs of the mild, moderate, and severe HIRI groups were also substantially higher than that of the sham group (8.23% ± 1.12%, *P* < 0.001). Therefore, mild and moderate HIRI were successfully identified by MB_neu_ targeted US imaging. The decrease in NID in the severe HIRI group might probably be because of filling defects caused by large patches of necrosis in the liver (Figure 7C). This result indicated that MB_neu_ was appropriate for evaluating mild to moderate HIRI, while routine CEUS was appropriate for evaluating severe HIRI. Furthermore, we identified a linear relationship (R^2^ = 0.9261) between pathological score and NID (Figure 7D). Moreover, we also identified a linear relationship (R^2^ = 0.8641) between ALT and NID (Figure 7E). All these results indicated that targeted US imaging using MB_neu_ could significantly improve the sensitivity of HIRI detection after liver transplantation compared with routine CEUS.

### Safety and immunogenicity of MB_neu_

**Figure 8:**
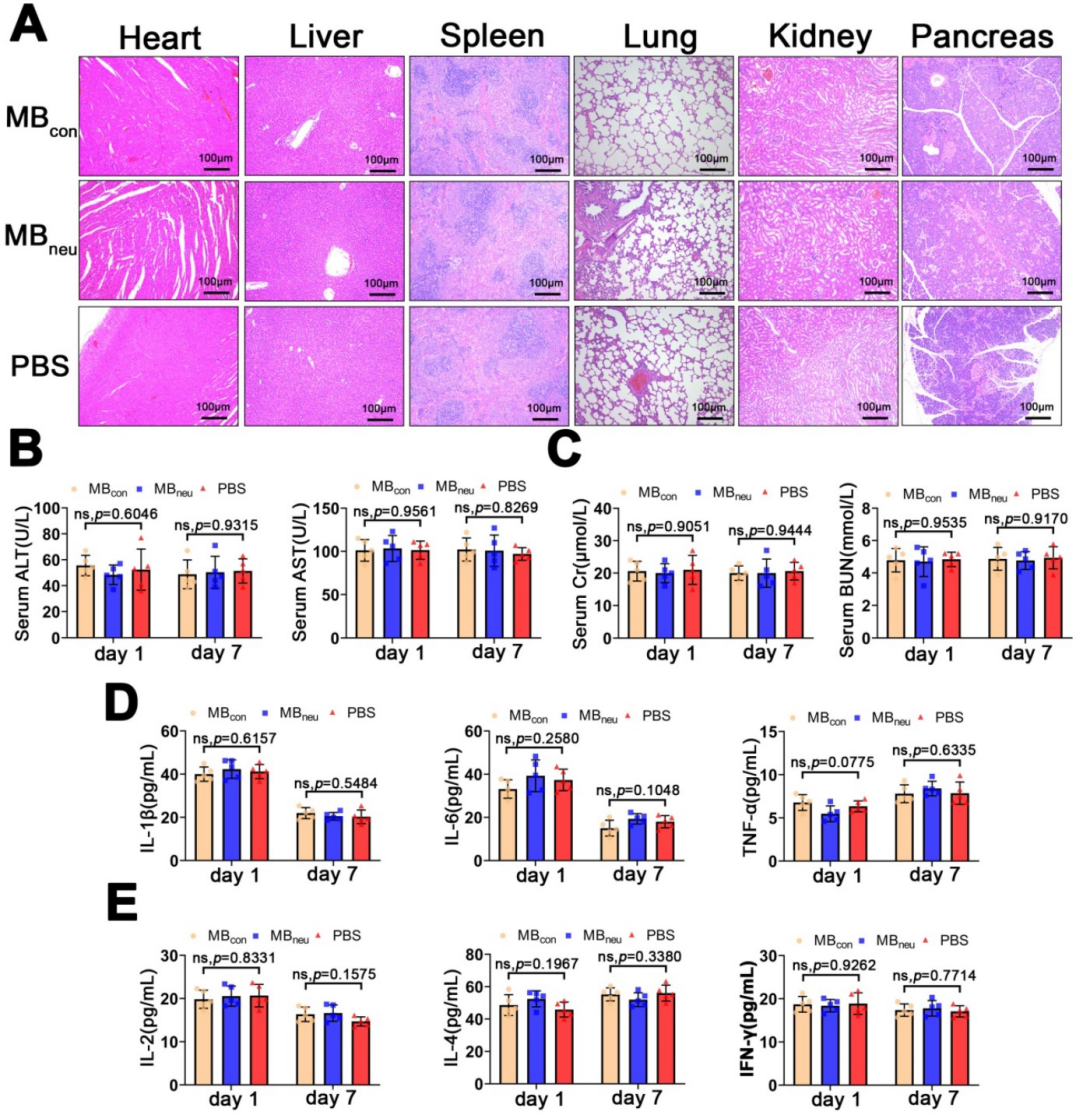
** Safety and immunogenicity of MB_neu_. (A)** Representative H&E-stained sections of major organs excised from rats 7 days after tail-vein injection of MB_con_, MB_neu_, or PBS (n = 5). The rats in the MB_neu_ group were treated with a high dosage of MB_neu_ (100 times the imaging dosage). **(B-E)** Serum levels of factors reflecting liver (ALT, AST) and kidney (Cr, BUN) function and main cytokines (IL-1β, IL-6, TNF-α, IL-2, IL-4, IFN-γ) in rats after tail-vein injection of MB_con_, MB_neu_, or PBS (n = 5). Blood samples were collected 1 and 7 days after MB or PBS intravenous administration.

The biosafety of MB_neu_ is of great concern. H&E staining of the major organs (heart, liver, spleen, lung, kidney, pancreas) after administration of MB_neu_ showed no observable pathological abnormalities or damage (Figure 8A). In addition, no significant differences were observed in serum biomarkers (AST, ALT, creatinine, blood urea nitrogen) and inflammatory cytokines (IL-1β, IL-6, TNF-α, IL-2, IL-4, IFN-γ) after injection of MB_neu_, MB_con_, or PBS (Figure 8B-E). These results suggested that MB_neu_ did not cause any tissue damage or inflammatory response and did not initiate any significant adaptive immune response and antibody production against membrane antigens.

### Distribution of MB_neu_

**Figure 9:**
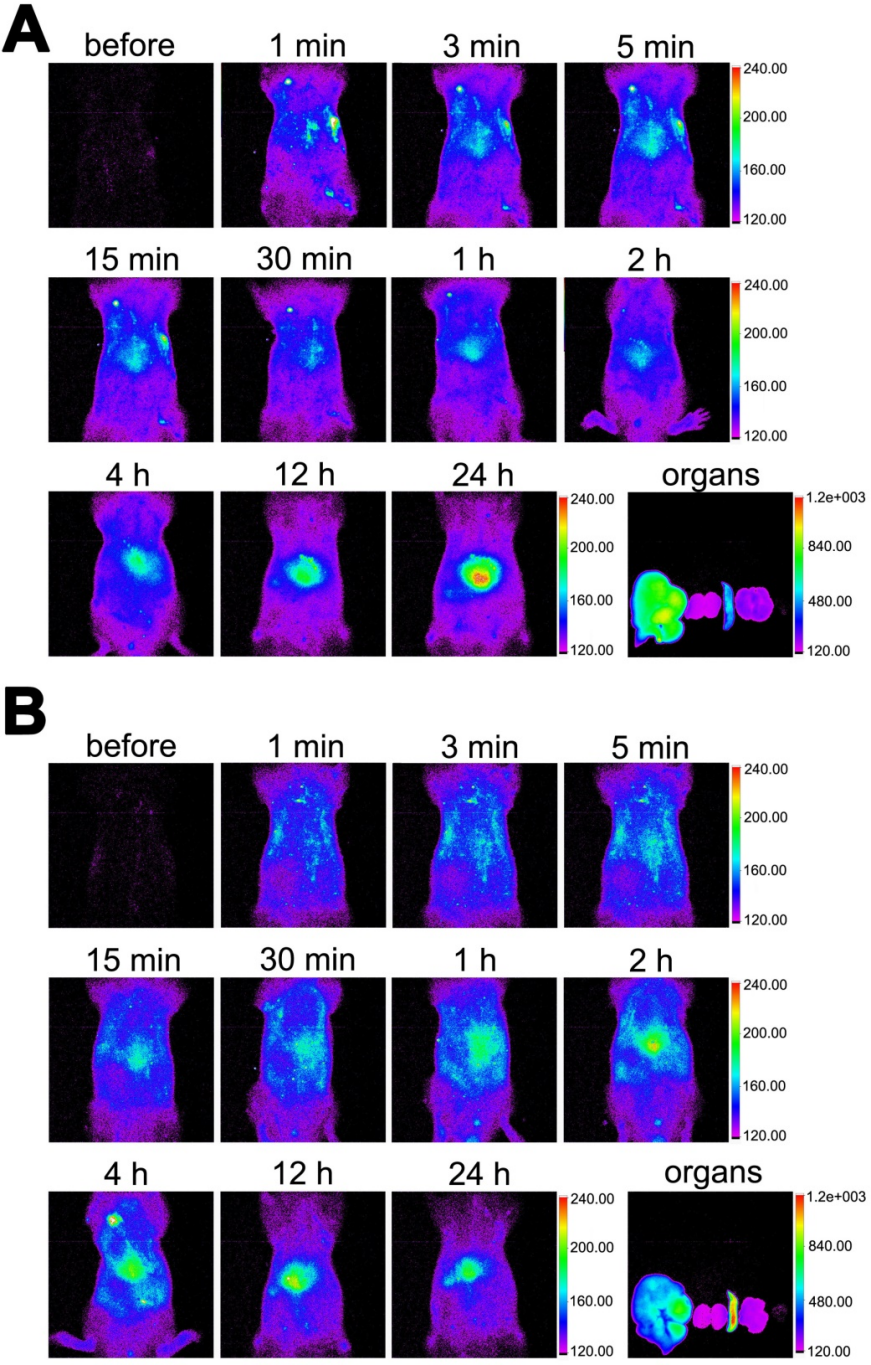
** Distribution of MB_neu_.** Fluorescence images of rats injected with DiR-labelled **(A)** MB_con_ or **(B)** MB_neu_. Organs from left to right: liver, kidneys, spleen, lung, heart.

As shown in Figure 9, the *in vivo* distribution of DiR-labelled MB_neu_ was similar to that of MB_con_. The fluorescence signal first distributed throughout the body and then concentrated in the liver and spleen. Unlike MB_con_, accumulation of MB_neu_ in the spleen was higher than in the liver. This difference may be because the cell membrane decreased the deformability of MB_neu_ compared with MB_con_.

## Discussion

Routine CEUS can be effectively used to observe patients with poor liver perfusion caused by HIRI 21, while molecular US imaging has the potential to quantitatively evaluate HIRI. Our previous research in this field achieved some progress 14. However, there are still some challenges that need to be addressed. First, single target UCAs (e.g., ICAM-1) often show low sensitivity because of their high diagnostic specificity, and a single adhesion molecule is insufficient to simulate the natural pathophysiological process. Moreover, adhesion to a single target is weaker than adhesion to multiple ones 22. Finally, immunogenicity caused by too many exogenous materials might limit clinical translation.

Neutrophils are major participants in the acute inflammatory response initiated by HIRI 23. During the reperfusion phase, a series of inflammatory mediators are produced, and the expressions of selectin and adhesion molecules are elevated. Subsequently, a large number of neutrophils are recruited by chemotaxis. The neutrophils roll on and firmly bind to the endothelium of hepatic sinusoidal vessels and then deform and migrate into liver tissue where they cause destruction 24. Neutrophil inflammatory infiltration usually reflects the severity of HIRI and is associated with poor prognosis in patients after OLT 25. In our study, we found that the expressions of adhesion molecules (ICAM-1, VCAM-1, E-selectin, P-selectin) increased to varying degrees in the early stage of HIRI and were positively correlated with HIRI. Several studies had previously shown that ICAM-1/selectin-targeted MBs in combination with CEUS were useful for estimating inflammation 14, 22, 26-28. A few studies have also developed acoustically responsive biomimetic vesicles for different theranostic applications, for example, targeting cerebral infarcts 17, 18. However, the shells of the MBs in these studies were entirely derived from cell membrane, which made the MBs unstable. Therefore, we took advantage of top-down and bottom-up strategies to incorporate fragments of neutrophil membrane with phospholipid liposomes to fabricate biomimetic neutrophil membrane-derived MBs (MB_neu_). These new UCAs retained membrane proteins derived from neutrophils, preferentially targeted inflamed hepatic tissues caused by HIRI, and had physicochemical properties typical of liposome-based MBs because of their artificial phospholipid content.

We used a modified destruction-replenishment method to acquire quantitative targeted US images. MB_neu_ was shown to be effective in identifying early HIRI and quantitatively estimating HIRI severity. We chose a partial HIRI model because we previously found that rats hardly survive a whole liver injury when the ischemia period was 60 min or longer 14. We would like to test the effectiveness and reliability of MB_neu_ in this extreme situation. In the severe HIRI group, large patches of necrosis with interrupted blood flow caused large filling defects in the CEUS images. MB_neu_ could not pass though this area, which led to a significant reduction in the imaging signal. This is probably the reason why NID was not correlated with severity of hepatic injury in the severe HIRI group. However, large filling defects in CEUS images themselves usually indicate poor prognosis. We also found that the liver injury score and ALT were linearly correlated with NID in mild to moderate injury. These results suggested that usage of MB_neu_ for molecular US imaging would be a reliable and precise method to quantitatively estimate HIRI in mild to moderate, but not severe, cases.

In this study, we developed a novel method to obtain biomimetic MBs that possess chemotaxis and adherence abilities similar to those of neutrophils. Cell membrane derived from neutrophils was integrated into a synthetic phospholipid bilayer to form a hybrid liposome, which was then mechanically vibrated with perfluoropropane to fabricate biomimetic MBs. Compared with the conventional method of synthesizing targeted MBs, our method has several advantages. First, it is a simple and convenient fabrication process that does not require complex chemical synthesis or purification steps. We believe this fabrication method has great potential for translational and clinical applications. Our biomimetic MBs contain two main components: synthetic phospholipids with high biological safety, and neutrophil membrane derived from the model animal. This new biological material was found to be safe, non-immunogenic, and effective in our study. All of these characteristics make clinical application convenient. Second, molecular US imaging and destruction-replenishment methods are becoming more advanced for clinical applications. Third, our fabrication method for obtaining biomimetic MBs can no doubt be applied to different clinical scenarios with different cell types in the future. For example, neutrophils as well as platelets play important roles in sepsis, so our biomimetic targeted MBs may help improve diagnosis. In summary, this new approach for synthesizing biomimetic UCAs for molecular US imaging quantified with an established CEUS technique has great clinical application potential, comparable to that of BR55 in patients with breast and ovarian lesions 29.

However, there are some limitations to this study. First, neutrophils were separated by density gradient centrifugation; therefore, some lymphocytes might have been included because of their similar particle size. In the future, we would like to improve the neutrophils isolation method, for example, by adopting immunoprecipitation techniques. We would also like to extend our fabrication method to other diseases using correlative cell membranes, for example, platelets for sepsis or oncocytes for tumors. Second, the relationship between NID and expression of ICAM-1, VCAM-1, E-selectin, and P-selectin was not analyzed in detail. Third, we did not investigate the therapeutic ability of MB_neu_, for instance, the potential for drug or gene delivery. It is our ambition to extend our new fabrication method to not only diagnosis but also response evaluation and treatment.

In conclusion, we developed a useful and effective method for fabricating a new type of biomimetic MB that is stable and efficient for the quantitative assessment of HIRI in rats by targeted CEUS imaging. This new type of MB may improve early diagnosis, prognosis, and therapeutic monitoring in patients suffering from HIRI. This new approach may also be applied to other diseases.

## Supplementary Material

Supplementary figures.

## Abbreviations

ALT: alanine transaminase
AST: aspartate transaminase
CEUS: contrast-enhanced ultrasound
DLS: dynamic light scattering
DPPC: 1,2-dipalmitoyl-*sn*-glycero-3-phosphocholine
DPPA: 1,2-dipalmitoyl-*sn*-glycero-3-phosphate
DSPE-PEG_2000_: 1,2-distearoyl-*sn*-glycero-3-phosphoethanolamine-*N*-[biotinyl(polyethylene glycol)_2000_]
H&E: hematoxylin/eosin staining
HIRI: hepatic ischemia-reperfusion injury
MBs: microbubbles
MB_con_: control microbubbles
MB_ery_: erythrocyte membrane-derived microbubbles
MB_neu_: neutrophil membrane-derived microbubbles
NID: normalized intensity difference
OLT: orthotopic liver transplantation
US: ultrasound
UCA: ultrasound contrast agent
