# Supplementary material for: Molecular ultrasound imaging of neutrophil membrane-derived biomimetic microbubbles for quantitative evaluation of hepatic ischemia-reperfusion injury
Source: Theranostics. 2021 May 8;11(14):6922–35. doi: 10.7150/thno.57794 (PMC8171082; doi:10.7150/thno.57794)
Supplement: Supplementary file 1 — Supplementary figures. [file thnov11p6922s1.pdf]

Molecular Ultrasound Imaging of Neutrophil Membrane-derived Biomimetic Microbubbles for Quantitative Evaluation of Hepatic Ischemia-reperfusion Injury

Zheng Zhang[1†], Xiaoyan Miao[2†], Weifeng Yao[1†], Jie Ren[2], Chaojin Chen[1], Xiang Li[1], Jing Yang[1], Yujia You[2], Yuejun Lin[2], Tinghui Yin[2*], Ziqing Hei[1*].

[1]Department of Anesthesiology, The Third Affiliated Hospital of Sun Yat-sen University, Guangzhou 510630, China.

[2]Department of Medical Ultrasonic, Laboratory of Novel Optoacoustic (Ultrasonic) imaging, The Third Affiliated Hospital of Sun Yat-sen University, Guangzhou 510630, China.

1. Supplementary Methods

1.1 Characterization of MBs

DLS measurements were collected at 25 °C on a 90 Plus/BI-MAS instrument (Brookhaven Instruments Corporation, USA). Three measurements for each sample were averaged.

To confirm the presence of neutrophil membrane in the MB_{neu} shell, the synthetic liposomes were fluorescently labeled by addition of 1μg DiO in the lipid mixture before liposome formation, and 1μg DiI was used to track the neutrophil membrane. Fluorescence images were acquired on an inverted fluorescence microscope (DMI 4000B, Leica, Germany).

To detect neutrophil membrane proteins in MB_{neu}, western blotting analysis was performed. 500 μL of MB_{neu}, neutrophil membrane (neutrosome), or neutrophil were lysed by addition of 200 μL of RIPA lysis buffer and 2 μL of phenylmethylsulfonyl fluoride (Solarbio, China) and incubated at 4 °C for 30 min. The mixture was then centrifuged at 2000 rcf for 2 min and the supernatant was collected. The concentration of protein in the supernatant was determined using a BCA kit (ThermoFisher, USA). Subsequently, protein samples (20 mg) were separated by 10% sodium dodecyl sulfate polyacrylamide gel electrophoresis and then transferred to polyvinylidene difluoride membranes. After blocking with 5% skim milk, the membranes were incubated overnight at 4 °C with rat antibodies against VLA-4, Mac-1, L-selectin, CD47, or Na^+/K^+ ATPase (1:1000 dilution; Abcam, USA). Horseradish peroxidase-conjugated goat anti-rabbit IgG (ProteinTech Group, Inc., USA) was used to amplify

the signal. Protein signals were detected using a chemiluminescence system (New Life Science Products, USA).

1.2 Immunohistochemistry

Liver sections were deparaffinized with xylene and alcohol and then incubated in 10 mM citrate buffer (pH 6.0) for 10 min at 90 °C. Then, 3% hydrogen peroxide in methanol was added to the sections at 4 °C for 30 min to inactivate endogenous peroxidases. The tissues were treated with 10% normal horse serum for antigen blocking for 1 h at room temperature and then incubated in rat primary antibodies against ICAM (1:2000 dilution), VCAM (1:500 dilution), P-selectin (1:200 dilution), or E-selectin (1:100 dilution) in 5% bovine serum albumin overnight at 4 °C. The sections were then washed with PBS three times and further incubated for 1 h with horseradish-conjugated goat anti-rabbit IgG secondary antibody. The immuno-reactivity on the tissue sections was visualized using the peroxidase substrate DAB. Finally, the nuclei were stained with hematoxylin.

1.3 Biosafety and immunogenicity of MB_{neu}

Healthy rats were anesthetized with 2% pentobarbital sodium (2 mL/kg i.p.) then randomly administered 500 μL of MB_{neu} or MB_{con} in PBS at a concentration of 1×10^8 MB/mL (100 times the imaging dosage) or the same volume of PBS via the tail vein (n = 5). Blood samples were collected 1 and 7 days after injection. The rats were sacrificed 7 days after injection and blood and major organs (heart, liver, spleen, lung,

kidney) were harvested to evaluate the biosafety of the MBs. Liver (AST, ALT) and kidney (Cr, BUN) function biomarkers in serum were measured using a clinical chemistry analyzer (Hitachi 7150). The concentration of cytokines (IL-1β, IL-6, TNF-α, IL-2, IL-4, IFN-γ) was determined by enzyme-linked immunosorbent assay (R&D Systems Inc., USA) according to the manufacturer's procedure. Organs were stained with H&E.

1.4 Distribution of MB_{neu}

Rats were anesthetized with 2% pentobarbital sodium (2 mL/kg i.p.) then administered 50 μL of DiR-labelled MB_{neu} or MB_{con} at a concentration of 1×10^7 MB/mL via the tail vein. The rats were imaged using an in vivo fluorescence imaging system (IVIS, PerkinElmer, USA) in luminescence imaging mode. Then the rats were sacrificed and the major organs (heart, liver, spleen, lung, kidney) were harvested for ex vivo imaging to determine the distribution of the MBs.

1.5 In vivo US imaging of MB circulation time

Targeted CEUS was performed using a clinical ultrasound imaging system (EPQ7 digital premium ultrasound system, PHILIPS, Netherlands) with a high-frequency linear array transducer operating with the following parameters: mechanical index, 0.08; frequency, 10 MHz; imaging depth, 3–4 cm. All imaging settings were kept constant throughout the imaging sessions for all animals. Healthy rats were administered 50 μL of MB_{con} or MB_{neu} in PBS at a concentration of 1×10^7 MB/mL via

the tail vein and then the MBs lifespan was recorded (n = 5).

2. Supplementary Results

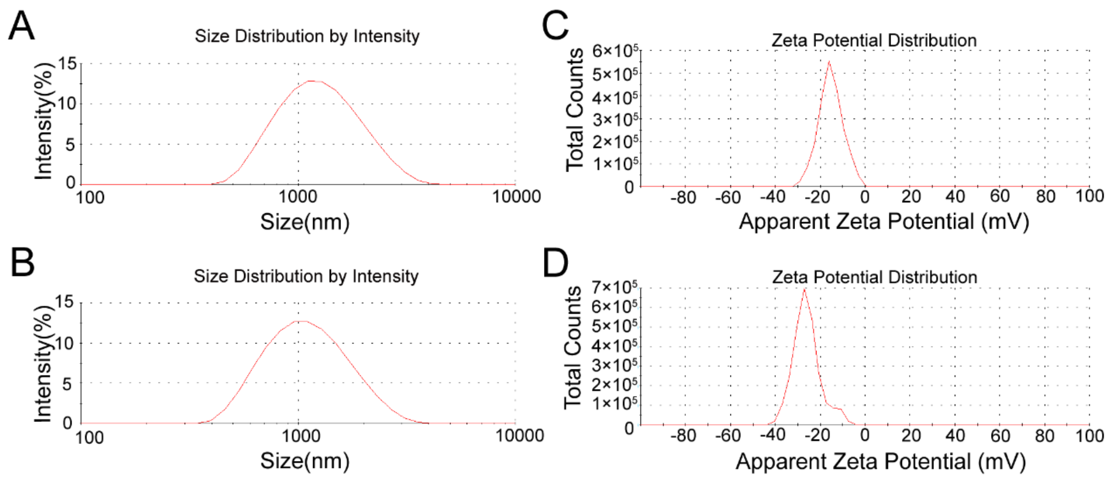

Fig. S1. DLS characterization of MB_{neu}. (A) Size distribution by intensity of MB_{con}. (B) Size distribution by intensity of MB_{neu}. (C) Zeta potential distribution of MB_{con}. (D) Zeta potential distribution of MB_{neu}. (n=3)

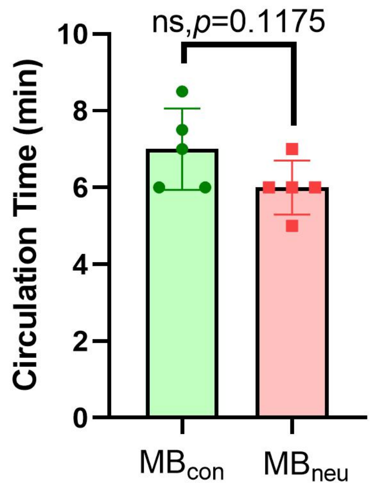

Fig. S2. Circulation time of MB_{neu} and MB_{con} (n = 5). The average blood circulation time of MB_{con} was 7 min, while that of MB_{neu} was ~6 min; however, there was no

statistically significant difference between the two groups. So, the circulation time of the newly developed MB_{neu} was ~6–7 min.
